# Direct LAMP for on-site rapid detection of *Vibrio parahaemolyticus* in shrimp (*Litopenaeus vannamei*) aquaculture water

**DOI:** 10.1371/journal.pone.0348231

**Published:** 2026-04-29

**Authors:** Xiaodan Pu, Feng Lu, Qianqian Yang, Zhenyu Han, Xueting Zhu, Jianlei Chen, Dahai Zhang, Xuzhi Zhang

**Affiliations:** 1 College of Food Science and Technology, Shanghai Ocean University, Shanghai, China; 2 State Key Laboratory of Mariculture Biobreeding and Sustainable Goods, Yellow Sea Fisheries Research Institute, Chinese Academy of Fishery Sciences, Qingdao, China; 3 Key Laboratory of Marine Chemistry Theory and Technology, Ministry of Education, Ocean University of China, Qingdao, China; 4 International Department, Qingdao No. 58 High School, Qingdao, China; University of Houston, UNITED STATES OF AMERICA

## Abstract

Effective on-site surveillance of pathogens in aquaculture environments via loop-mediated isothermal amplification (LAMP) remains challenging due to the impractical and time-intensive DNA extraction required in field settings. In this study, a DNA extraction-free approach, i.e., direct LAMP (dLAMP), was evaluated, additionally concerning key physicochemical inhibitors derived from real, complex aquaculture water matrices. Eight representative water samples were collected from *Litopenaeus vannamei* aquaculture systems, encompassing broad ranges of salinity (7.61‰–32.88‰), pH (6.98–8.41), chemical oxygen demand (COD: 6.00–12.25 mg/L), soluble reactive phosphate (SRP: 0.13–1.24 mg/L), dissolved inorganic nitrogen (DIN: 0.67–3.09 mg/L), and total suspended solids (TSS: 133–737 mg/L). Boiled aquaculture water samples served directly as DNA templates for the LAMP detection of *Vibrio parahaemolyticus* with *tlh* gene. While pH, COD, SRP, DIN, and TSS had negligible effects on amplification efficiency, elevated salinity was found to significantly reduce assay sensitivity. Adjusting the salinity using deionized water to ≤ 10.00‰ effectively mitigates its inhibitory effect. Under optimized conditions, 10 µL of boiled aquaculture water was sufficient for reliable detection without compromising sensitivity or specificity. The detection limit was determined to be 10^2^ CFU/mL (with a detection probability of 67%). Using DNA dye GeneFinder™ to show amplicon visually, the entire workflow, including sampling, salinity adjustment (if needed), boiling, chilling, and isothermal incubation, required no more than 1 h. This dLAMP method requires no specialized equipment beyond a portable heater, demonstrating a strong potential for point-of-care applications, and enabling practical field surveillance of pathogens in aquaculture water.

## Introduction

*Litopenaeus vannamei* (also known as Pacific white shrimp) is the dominant species in global shrimp aquaculture, accounting for the majority of production [1,2]. However, this species is highly susceptible to waterborne pathogens, which frequently cause significant yield losses and economic damage to the aquaculture industry [2]. Effective surveillance of pathogens in aquaculture environments is therefore critical for implementing timely and targeted disease control strategies [3].

Conventional culture-based methods, such as the classical plate counting, remain the gold standard for pathogen detection in aquaculture settings [4,5]. However, their requirement for over 20 h of incubation renders them unsuitable for rapid detection purposes. Even the electronic microbial growth analyzer-based method, which enables direct quantification of viable *Vibrio harvey*i pathogens on-site in aquaculture facilities, typically requires more than 4 h [6]. To address the urgent need for timely pathogen surveillance, recently, researchers have developed various rapid detection technologies [7]. For instance, gene amplification-based techniques, including polymerase chain reaction (PCR), quantitative PCR (qPCR), and loop-mediated isothermal amplification (LAMP), have emerged as promising tools for rapid pathogen detection by bypassing the cultivation step [8], thereby enhancing the surveillance efficiency. Among these, LAMP stands out for its high sensitivity, specificity, and minimal equipment requirements [9]. Compared with PCR and qPCR, LAMP also demonstrates superior tolerance to coexisting impurities such as non-target DNA, cell debris, blood, serum, and food ingredients [10].

Several studies have demonstrated the feasibility of using boiled microbial suspensions to directly serve as DNA templates for LAMP assay without DNA extraction. For example, Sriworarat et al. [11] reported that the supernatant of boiled *Leishmania siamensis* samples could serve as DNA templates for LAMP. Sayad et al*.* [12] found that the supernatant of heated *Salmonella* suspensions could be directly used as DNA templates for LAMP. Tian et al*.* [13] further confirmed that bacterial DNA released by heating could be directly subjected to LAMP reaction system. Undoubtedly, the elimination of steps such as bacterial isolation, purification, cell lysis, and DNA extraction significantly accelerates the detection process, making direct LAMP (dLAMP) a promising tool for point-of-care applications [7,8,14].

If boiled aquaculture water could be directly added to the LAMP reaction mixture to serve as DNA templates for detecting target pathogens, this approach would offer a highly promising strategy for on-site microbial safety surveillance. However, previous studies on dLAMP assays have predominantly been conducted using bacterial suspensions in simple laboratory matrices (e.g., pure water, buffer solutions, or culture media) [12,13,15]. When target pathogens are present in complex matrices such as biological samples or food, centrifugation has been required after boiling treatment to collect the supernatant for the use as the DNA template [11,12,16]. Unlike simple laboratory media, aquaculture water, particularly in *Litopenaeus vannamei* ponds, typically exhibits complex physicochemical characteristics, including variable salinity, pH, organic matter, nutrients, and suspended solids [17]. Theoretically, complex ionic interactions may present a unique challenge for *Bst* DNA polymerase that simplified laboratory models cannot replicate. However, whether these complex physicochemical factors will interfere with LAMP-based detection when boiled aquaculture water used directly as DNA templates has not been systematically investigated.

In this study, representative aquaculture water samples were collected from eight *Litopenaeus vannamei* systems. Their physicochemical properties, including salinity, pH, chemical oxygen demand (COD), soluble reactive phosphate (SRP), dissolved inorganic nitrogen (DIN), and total suspended solids (TSS), were systematically characterized. Using the thermolabile hemolysin (*tlh*) gene as a specific marker for *Vibrio parahaemolyticus* (*V. parahaemolyticus*), a dLAMP assay was developed for targeted detection of this pathogen. Pure extracted genomic DNA and crude DNA mixture prepared by boiling method served as templates for LAMP assays in comparison. The primary objective of this study was to clarify the effect of physicochemical factors originating from aquaculture water on the dLAMP assay, and further to develop a practical, and field-deployable method for rapidly screening pathogens in aquaculture water.

## Materials and methods

### Bacterial strains and chemicals

Standard bacterial strains of *V. parahaemolyticus* ATCC 17802, *V. parahaemolyticus* ATCC 33847, *V. parahaemolyticus* ATCC 27969, *Escherichia coli* ATCC 43888, *Escherichia coli* ATCC 35150, *Enterobacter aerogenes* BNCC 360397, *Staphylococcus aureus* ATCC 25923, *Lactobacillus bulgaricus* BNCC 187941, *Salmonella Typhimurium* CMCC(B) 50115, *Pseudomonas aeruginosa* ATCC 27853, *Listeria monocytogenes* ATCC 15313, *Vibrio vulnificus* BNCC 186281, *Vibrio vulnificus* ATCC 27562, *Vibrio alginolyticus* ATCC 33787, *Vibrio harveyi* ATCC BAA-1117, *Vibrio anguillarum* ATCC 19264, *Vibrio tubiashii* ATCC 19109, *Vibrio cholerae* ATCC 51395, and *Vibrio fluvialis* ATCC 33809, were purchased from BIOBW Biotechnology Co., Ltd. (Beijing, China). They were cultured following the protocols described previously [6,18] with minor modifications. Briefly, *V. parahaemolyticus* was seeded onto 2216E agar and incubated overnight in an incubator (RADOBIO Scientific Co., Ltd., Shanghai, China) with constant shaking at 36°C. Active strains were then selected and further transferred to fresh liquid thiosulfate citrate bile salts sucrose (TCBS) broths. After a second incubation for ~12 h to achieve mid-exponential phase, cells were harvested by centrifugation. Briefly, TCBS broths were centrifuged at 5000 × *g* for 15 min at 4°C using a 5810R Eppendorf centrifuge to isolate *V. parahaemolyticus* cells. The pelleted biomass was then re-suspended to constitute a series of bacterial suspensions with gradient concentrations. Cell concentrations were measured using either the colony-forming unit (CFU) counting method or optical density (OD) method as reported previously [19]. Other bacterial strains were cultured with Luria-Bertani (LB) broth unless stated otherwise.

All culture media, including 2216E agar, TCBS agar, LB broth, and TCBS broth, were acquired from Hope Bio-Technology Co., Ltd. (Qingdao, China). Genomic DNA extraction and purification were performed using a Bacteria Genomic DNA Extraction Kit (Tiangen Biotech Co., Ltd., Beijing, China). The fluorescent DNA dye GeneFinder™ was purchased from Biov Co., Ltd. (Xiamen, China). Electrophoresis reagents, including loading buffer, agarose, the DNA dye GelGreen, and DL2000 DNA marker, were purchased from Takara Co., Ltd. (Dalian, China). Enzymes and biochemicals, namely deoxynucleotide triphosphates (dNTPs), betaine, magnesium sulfate (MgSO₄), and *Bst* 2.0 WarmStart DNA polymerase, were purchased from New England Biolabs (NEB; Ipswich, MA, USA). Primers targeting the *tlh* gene of *V. parahaemolyticus* [20] were custom-synthesized by Sangon Biotech (Shanghai) Co., Ltd. (Shanghai, China); their sequences are provided in Table 1. All other routine chemicals (e.g., sodium chloride [NaCl], hydrochloric acid [HCl], sodium hydroxide [NaOH], disodium phosphate [Na₂HPO₄], ammonium nitrate [NH₄NO₃], and humic acid) were of analytical grade and supplied by Sinopharm Chemical Reagent Co., Ltd. (Shanghai, China).

**Table 1:** Primers used for specifically recognizing the *tlh* gene of *V. parahaemolyticus* by LAMP.

Primer	Sequence (5’-3’)
**F3**	CGGTGACAGCTTGTCTGA
**B3**	TCACCAACCCCTGTTAGC
**FIP**	CCTAAGAACCAGCTGTTCGGGTTTTACAGGCAACATCTTTAACGC
**BIP**	TTGCCAAAGCGAAGAACCTTCCTTTTTATTGGTTCTCACCAGCCG
**LF**	AGGGAAGCGCCATTGTG
**LB**	GCTCTACAACTGGGCAGT

### Collection and pretreatment of aquaculture water samples

Eight aquaculture water samples were collected from distinct aquaculture farms and plants. The sampling sites are presented in Table 2. The geographic coordinates (latitude and longitude) of the sampling sites were measured in situ using a handheld GNSS receiver (i70, CHC Navigation, Shanghai, China). Water was collected approximately 20 cm below the surface using a polyethylene sampler and stored in amber glass bottles. To establish a rigorous baseline for evaluating the inhibitory effects of physicochemical factors on the dLAMP assay via the standard addition method, it was essential to eliminate any biological background noise that could compromise quantitative accuracy. The pre-existing *V. parahaemolyticus* and extracellular DNA in raw aquaculture water would lead to an overestimation of the spiked bacterial concentration, thus invalidating the quantitative evaluation of the assay’s sensitivity. Unless stated otherwise, samples were sequentially sterilized at 121°C for 30 min to eliminate native microorganisms [18], and then subjected to UV irradiation (120 mJ/cm²) for 10 min to degrade extracellular DNA [21]. The treated samples, designated as TS1 through TS8, were subsequently stored in the dark at room temperature. Prior to use, samples were vortexed to re-suspend any settled particulate matter.

**Table 2:** Collection sites of aquaculture water samples.

Sample	Sampling site	Coordinates
**S1**	Haiyang Huanghai Aquaculture Co., Ltd	36.6817°N, 121.1587°E
**S2**	Huangdao Haiqing Aquaculture Co., Ltd	36.0435°N, 120.2254°E
**S3**	Shandong Evergreen Seed Technology Co., Ltd	37.1414°N, 118.7509°E
**S4**	Yellow Sea Fisheries Research Institute	36.0771°N, 120.3865°E
**S5**	Rizhao Xinhui Aquatic Seedling Co., Ltd	35.4169°N, 119.5268°E
**S6**	Qingdao Blue-seed Research Institute	36.3902°N,120.4476°E
**S7**	Jimo Dingziwan Aquaculture Co., Ltd	36.3817°N, 120.6822°E
**S8**	Langya R&D Base of the YSFRI	35.6032°N, 119.6242°E

### Measurements of physicochemical properties of aquaculture water samples

Prior to the measurement of their physical and chemical properties, these treated water samples, TS1 through TS8, were vortexed to re-suspend settled particulate matters. Salinity and pH of each sample were measured at room temperature using a 556 Handheld Multi-parameter Instrument (YSI Incorporated, Yellow Springs, USA). COD was measured according to the Chinese Ministry of Environmental Protection standard protocol (HJ 828–2017). SRP, ammonium (NH₄ ⁺ -N), nitrite (NO₂ ⁻ -N), and nitrate (NO₃ ⁻ -N) concentrations were determined by the molybdate blue method, indophenol-blue method, α-naphthylamine method, and the UV-spectrophotometry method, respectively [22]. DIN was calculated as the sum of NH₄ ⁺ -N, NO₂ ⁻ -N, and NO₃ ⁻ -N. TSS was determined using the protocol reported by Das et al. [23]. Briefly, 1.0 L aliquots were filtered through pre-weighed 0.7 μm glass microfiber filters (GF/F, 47 mm; Whatman), and the dry mass was determined using an MS204/A electronic balance (METTLER TOLEDO).

### Preparation of DNA template for LAMP

#### Extracted genomic DNA.

The genomic DNA of *V. parahaemolyticus* was extracted and purified using a Bacteria Genomic DNA Extraction Kit according to the manufacturer’s instructions. The concentration of extracted DNA was quantified with a UV spectrophotometer (Thermo Fisher Scientific, Waltham, MA, USA), and the purity was evaluated by determining the ratio of the absorbance at 260 nm to 280 nm [24]. Subsequently, purified genomic DNA was aliquoted and stored at −20°C. Unless stated otherwise, the storage duration did not exceed 2 months. This purified genomic DNA dissolved in water was designated as “extracted genomic DNA”.

#### Crude DNA mixture.

Crude DNA was prepared using a boiling method adapted from Sun et al. [16] with minor modifications. Briefly, bacterial cells re-suspended in ultrapure water at concentrations of 10^7^, 10^6^, 10^5^, 10^4^, 10^3^, 10^2^, and 10^1^ CFU/mL were heated at 100°C for 5 min and immediately chilled in an ice-water bath. The resulting suspensions were designated as crude DNA. For the preparation of crude DNA in aquaculture water, bacterial cell-free and DNA-free water samples (TS1–TS8) replaced ultrapure water. Prior to serving as templates for LAMP assays, these crude DNA mixtures were homogenized by manual shaking rather than centrifugation as described by Sun et al. [16].

To investigate the effect of salinity originating from aquaculture water on the LAMP assay, NaCl was added to TS2 samples to achieve salinities of 10.00‰, 13.00‰, 16.00‰, 19.00‰, 21.00‰, and 24.00‰ (denoted as modified TS2 samples). Similarly, HCl was used to adjust pH in TS7 samples, while humic acid, lyophilized marine sediment powder, Na₂HPO₄, and NH₄NO₃ were used to modify concentrations of COD, TSS, SRP, and DIN, respectively, in pretreated aquaculture water samples. Subsequently, these adjusted TS matrices were used to prepare crude DNA in adjusted aquaculture water using the aforementioned boiling method.

### LAMP system and product analysis

The LAMP reactions were carried out referring to the protocol reported by Benjakul et al*.* [20] with minor modifications. Each 100 μL reaction mixture contained the following components: 0.2 µM each of F3 and B3 primers, 0.4 µM each of LoopF and LoopB primers, 1.6 µM each of FIP and BIP primers, 1.6 mM of dNTPs, 0.8 mM of betaine, 5 mM of MgSO₄, and 1.6 U of *Bst* 2.0 WarmStart DNA polymerase. In this study, 10 μL extracted genomic DNA solution, and crude DNA mixture (in water, aquaculture water, or adjusted aquaculture water), were used to serve as DNA templates for LAMP assays. Unless stated otherwise, extracted genomic DNA and sterilized pure water served as the positive and negative controls, respectively. All reactions were incubated in a thermal cycler (Biorad, Temecula, USA) at 62°C for 40 min. Amplification products, namely amplicons, were analyzed by electrophoresis on 2% agarose gels using an imaging system (MF-ChemiBis 3.2, DNR Bio-Imaging Systems, Israel) and electrophoresis apparatus (DY-6, Xinghua Apparatus Ltd., China). Additionally, products were visualized using fluorescent DNA dye GeneFinder™ as reported previously [25,26].

### Sensitivity

The re-suspended *V. parahaemolyticus* cells in water (10^7^ CFU/mL) was divided into two aliquots. One aliquot was used for genomic DNA extraction following the protocol as described above. The other aliquot was boiled to make crude DNA in aquaculture water or adjusted aquaculture water as described above. Ten-fold serial dilutions of the extract genomic DNA were prepared using nuclease-free water. Similarly, ten-fold serial dilutions of crude DNA were prepared using TS1, TS2, TS3, and other adjusted aquaculture water matrices. Subsequently, 10 μL of each dilution series was added to LAMP reaction mixtures as DNA templates. All LAMP assays were performed under identical conditions as described above.

### Specificity

When the crude DNA in aquaculture water or adjusted aquaculture water (salinity ≤ 10.00‰,) was used as DNA templates for LAMP assays, the specificity was evaluated with eight non-*Vibrio* strains (e.g., *Escherichia coli*, *S. aureus*) and ten *Vibrio* strains (e.g., *V. vulnificus*, *V. harveyi*). All bacterial strains were cultured in broth medium and harvested from their broths by centrifugation [27]. These bacterial cells were washed with sterile water for three times. Then the pelleted biomass was transferred into TS1, TS2, TS3, and other adjusted aquaculture water to prepare inoculated samples. Subsequently, these inoculated samples were used to prepare crude DNA in aquaculture water and adjusted aquaculture water using the boiling method detailed as described above.

### Statistical analysis

Unless stated otherwise, all trials were conducted in triplicate. Data were subjected to statistical analysis using IBM SPSS Statistics (Version 21) and are presented as mean values or mean ± standard deviation (SD) of replicates. Pearson correlation matrices were generated computationally using the statistical toolkit in OriginPro 2022 (OriginLab Corporation, Northampton, MA, USA). The assumptions of linearity and normality were verified using scatter plots and the Shapiro-Wilk test, respectively, prior to Pearson correlation analysis.

## Results

### Results of LAMP

**Fig 1:**
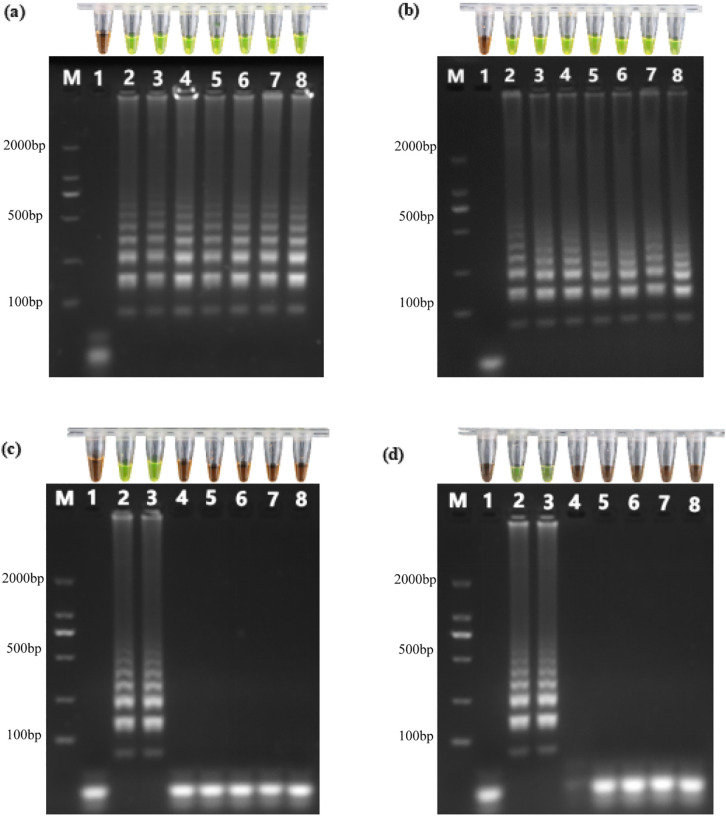
Extracted genomic DNA in water (a), crude DNA in water (b), extracted genomic DNA in TS1 through TS8 (c), and crude DNA in TS1 through TS8 (d), respectively, served as templates for LAMP to detect the *tlh* gene of *V. parahaemolyticus*. Resulting amplicons were analyzed by gel electrophoresis and the fluorescent DNA dye GeneFinder™. Panel (a): Lane 1: negative control; Lanes 2–8: extracted genomic DNA served as template was extracted from 10^6^, 10^5^, 10^4^, 10^3^, 10^2^, 10^1^, and 1^00^ CFU/µL bacterial cells, respectively. Panel (b): Lane 1: negative control; Lanes 2–8: boiled bacterial suspensions of 10^6^, 10^5^, 10^4^, 10^3^, 10^2^, 10^1^, and 1^00^ CFU/µL, respectively, served as DNA template. PCR tubes aligned above each gel electrophoresis lane correspond to visualization results obtained upon addition of GeneFinder™.

Fig 1a displays the electrophoretic profiles of LAMP products employing extracted genomic DNA to serve as templates. In the negative control (Lane 1), no amplicons were detected. In contrast, Lane 2 to Lane 8 exhibited reproducible ladder-like banding patterns [11,20], indicative of successful amplification through the formation of varying-length amplicons. Concurrently, as expected, after the addition of 2 μL DNA dye GeneFinder™, the colour of these liquid mixtures changed from orange to green [25,26].

Fig 1b presents the gel electrophoresis and visualization results of dLAMP products employing crude DNA mixture to serve as templates. The negative control (Lane 1) exhibited no ladder-like banding pattern. Consistent with this observation, no color change to green occurred in the corresponding dLAMP mixture following the addition of DNA-binding dye GeneFinder™. In contrast, Lanes 2–8 demonstrated reproducible ladder-like electrophoretic profiles. Concurrently, the reaction mixtures in these tubes underwent a distinct color change from orange to green upon the addition of GeneFinder™, further confirming successful amplification.

Genomic DNA was extracted from 10^7^ CFU of *V. parahaemolyticus* using a Bacterial Genomic DNA Extraction Kit. A 10 μL aliquot of the purified genomic DNA solution was introduced into 1 mL aliquots of TS1 through TS8, respectively. Subsequently, 10 μL portions of these DNA-spiked samples served as templates for LAMP assays. Fig 1c displays the electrophoretic profiles and visualization results of amplicons. When DNA-spiked TS2 and TS3 samples were used as templates, reproducible ladder-like amplification patterns were observed. Correspondingly, post-reaction mixtures in PCR tubes exhibited a color change from orange to green upon the addition of GeneFinder™, indicating successful amplification. In contrast, LAMP reactions using the rest of DNA-spiked samples (TS1, TS4–TS8) to serve as template yielded no ladder-like amplicons. Correspondingly, no color change occurred following the addition of GeneFinder™, denoting failed LAMP reactions. The dLAMP assay failed to yield positive signals in the majority of real aquaculture water samples. This finding led us to hypothesize that certain physicochemical components derived from the complex aquaculture matrix exerted a strong inhibitory effect on the *Bst* DNA polymerase. Consequently, a systematic evaluation of various factors, including salinity, pH, and organic load, was conducted to pinpoint the specific inhibitors responsible for this interference.

Two hundred microliters of an aqueous bacterial suspension (10^7^ CFU/mL) was added to 100 mL each of TS1 through TS8 to make a final concentration of 10^4^ CFU/mL. Subsequently, these bacteria-spiked samples were boiled, followed by rapid chilling, to prepare crude DNA mixtures. A 10 μL aliquot of each resulting mixture was added into LAMP system to serve as DNA templates. As shown in Fig 1d, successful amplification occurred when crude DNA-spiked TS2 and TS3 samples were used as templates, whereas reactions failed with the rest of samples (TS1, TS4–TS8). We increasingly hypothesized that inhibitory effects on the dLAMP reaction might arise from one or more physicochemical factors inherent to TS1, and TS4–TS8.

### Physicochemical properties of treated aquaculture water samples

Salinity, pH, COD, TSS, SRP, and DIN are key physicochemical parameters routinely concerned in *Litopenaeus vannamei* aquaculture [28]. Table 3 presents these parameters for eight treated aquaculture water samples (TS1 to TS8). Salinity exhibits a range spanning from 7.61‰–32.88‰. The pH values fluctuate within the interval of 6.98 to 8.41. The COD displays a variation from 6.00 mg/L to 12.25 mg/L. The concentration of the TSS varies between 133 mg/L and 737 mg/L. The SRP varies over the range of 133.62–1240.23 µg/L. Regarding the DIN, its concentration ranges from 665.91 to 3091.67 µg/L. These data fall well within the normal range specified for the water quality requirements in the aquaculture of *Litopenaeus vannamei* [17,28].

**Table 3:** Physicochemical parameters (mean value, *n* = 3) of treated aquaculture water samples.

Sample	Salinity (‰)	pH	COD (mg/L)	TSS (mg/L)	SRP (µg/L)	DIN (µg/L)
**TS1**	30.86	8.09	12.25	220	848.83	3091.67
**TS2**	7.61	7.87	11.85	432	1085.36	946.08
**TS3**	11.62	7.24	6.00	259	1141.68	934.35
**TS4**	30.01	6.98	8.35	133	360.29	665.91
**TS5**	29.44	8.17	10.10	219	1240.23	1636.71
**TS6**	32.88	8.10	8.35	737	847.42	1057.22
**TS7**	29.55	8.41	6.60	292	547.54	1453.44
**TS8**	23.15	8.21	10.10	255	133.62	2356.39

### Pearson correlation matrix analysis

**Fig 2:**
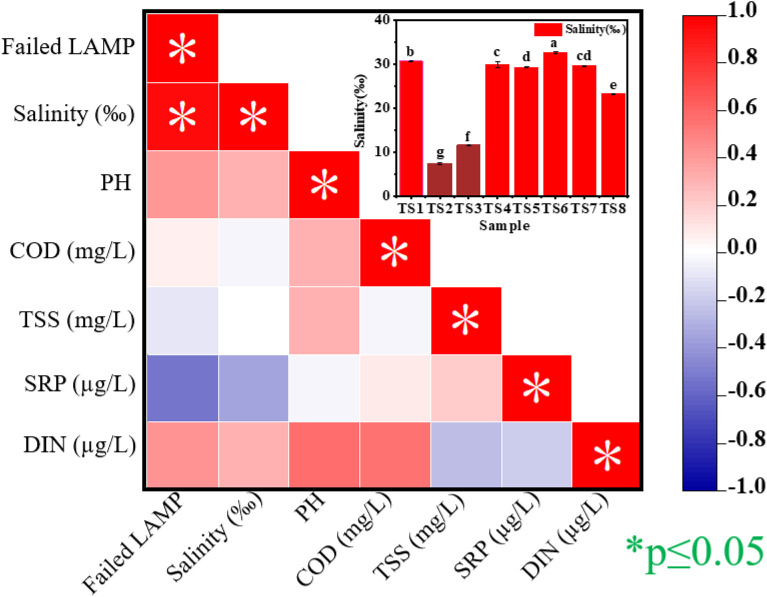
Pearson correlation matrix between all pairwise combinations of properties, as well as between failed LAMP reactions and individual variables. Within the inset, values denote mean salinity levels (*n* = 3), with error bars representing the SD of these means. Significant differences (*P* < 0.05) are indicated by distinct superscript letters above bars.

TS1 through TS8 exhibited variations in salinity, pH, COD, TSS, SRP, and DIN parameters. To identify potential inhibitory factors for the LAMP reaction, Pearson correlation analysis was employed to assess relationships between these physicochemical parameters and reaction failure rates. The Pearson correlation coefficient matrix, defined as the covariance of two variables divided by the product of their standard deviations [29], revealed a strong positive correlation between salinity and LAMP reaction failure (r = 1.0; Fig 2). In contrast, pH, COD, TSS, SRP, and DIN demonstrated negligible correlations with reaction failure (r ≤ 0.4). These results indicate that elevated salinity may inhibit LAMP reactions. It is worth noting that while a Pearson correlation coefficient of r = 1.0 was observed, this result should be interpreted with caution. Given the limited sample size (*n* = 8), this perfect correlation may not fully capture the inherent variability of complex aquaculture environments, and further validation with a larger number of samples is warranted to confirm these findings.

### Effect of salinity on the LAMP

Among TS1 through TS8, TS2 exhibited the lowest salinity. A series of measured quantities of NaCl were introduced to six aliquots of TS2 samples to prepare modified samples with salinities of 10.00‰, 13.00‰, 16.00‰, 19.00‰, 21.00‰, and 24.00‰, respectively. Extracted genomic DNA of *V. parahaemolyticus* was spiked into both the TS2 samples and the prepared modified samples to achieve a final DNA concentration of 5 pg/mL in each sample. Concurrently, *V. parahaemolyticus* were spiked into a separate group of TS2 sample and salinity-modified samples to attain 10^5^ CFU/mL. This latter group was subsequently subjected to boiling lysis to prepare crude DNA mixtures. These extracted genomic DNA and crude DNA-spiked TS2 and modified samples, served as templates for LAMP reactions. Resulting amplicons were detected using both gel electrophoresis and DNA dye GeneFinder™.

**Fig 3:**
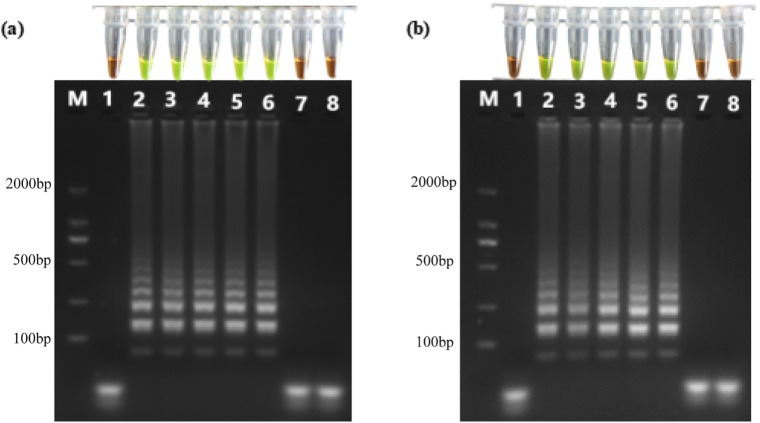
Extracted genomic DNA (a) and crude DNA (b) in TS2 and modified TS2 served as DNA templates for LAMP assays to detect the *tlh* gene of *V. parahaemolyticus.* Lane 1: negative control; Lanes 2–8: salinity of 7.61‰, 10.00‰, 13.00‰, 16.00‰, 19.00‰, 21.00‰, and 24.00‰, respectively. PCR tubes aligned above each gel electrophoresis lane correspond to visualization results obtained upon addition of GeneFinder™.

Fig 3a presents LAMP results using extracted genomic DNA-spiked TS2 and extracted genomic DNA-spiked modified TS2 samples as templates. Reproducible ladder-like amplicon patterns were observed when salinities of TS2 and modified TS2 samples were 7.61‰, 10.00‰, 13.00‰, 16.00‰, and 19.00‰. Correspondingly, post-reaction mixtures in PCR tubes exhibited a color change from orange to green upon the addition of DNA dye GeneFinder™, indicating successful amplification. Conversely, when salinities of modified TS2 samples were 21.00‰, and 24.00‰, no ladder-like patterns of amplicons were observed; and no color change occurred after the addition of GeneFinder™, demonstrating failed amplification. Fig 3b displays analogous results obtained with crude DNA-spiked TS2 and modified TS2 samples as templates. Consistent with the above findings, LAMP reactions proceeded successfully at modified TS2 with salinities ≤ 19.00‰, but failed at salinities of 21.00‰ and 24.00‰.

Furthermore, TS1, TS4 through TS8 were mixed with deionized water at equal volumes (1:1 v/v) to reduce salinity. *V. parahaemolyticus* was spiked into these diluted samples to achieve a final concentration of 10^5^ CFU/mL. Subsequently, boiled method was employed to prepare crude DNA in these modified aquaculture water samples. Two microliters of each resultant mixture served as DNA templates for LAMP assays. Results of the gel electrophoresis analysis indicated successful amplification in all reactions.

### Sensitivity

*V. parahaemolyticus* was introduced into both TS2 and salinity-modified TS2 (to be 10.00‰, 13.00‰, 16.00‰, and 19.00‰) to achieve final concentrations of 1^00^ CFU/mL, 10^1^ CFU/mL, 10^2^ CFU/mL, 10^3^ CFU/mL, 10^4^ CFU/mL, and 10^5^ CFU/mL. All inoculated samples were subjected to boiling followed by immediate chilling. Subsequently, 10 µL aliquots of the resultant crude DNA-containing samples served as templates for dLAMP assays. Amplicons were visually characterized using DNA dye GeneFinder™. Results are detailed in Table 4.

**Table 4:** Visualization results of dLAMP assays using 10 µL boiled TS2 and salinity-modified TS2 containing various amount of *V. parahaemolyticus* to serve as DNA templates (*n* = 3).

Bacteria concentration (CFU/mL)	TS2 (7.61‰)^a^	Modified TS2 (10.00‰) ^a^	Modified TS2 (13.00‰) ^a^	Modified TS2 (16.00‰) ^a^	Modified TS2 (19.00‰) ^a^
**10** ^**0**^	(-)^b^ (-) (-)	(-) (-) (-)	(-) (-) (-)	(-) (-) (-)	(-) (-) (-)
**10** ^**1**^	(-) (-) (+)^c^	(-) (-) (-)	(-) (-) (-)	(-) (-) (-)	(-) (-) (-)
**10** ^**2**^	(-) (+) (+)	(-) (-) (+)	(-) (-) (-)	(-) (-) (-)	(-) (-) (-)
**10** ^**3**^	(+) (+) (+)	(+) (+) (+)	(-) (+) (+)	(-) (-) (+)	(-) (-) (-)
**10** ^**4**^	(+) (+) (+)	(+) (+) (+)	(+) (+) (+)	(-) (+) (+)	(-) (-) (+)
**10** ^**5**^	(+) (+) (+)	(+) (+) (+)	(+) (+) (+)	(+) (+) (+)	(+) (+) (-)

(‰)^a^- salinity. (-)^b^ – failed reaction. (+)^c^ – successful reaction.

### Specificity

**Fig 4:**
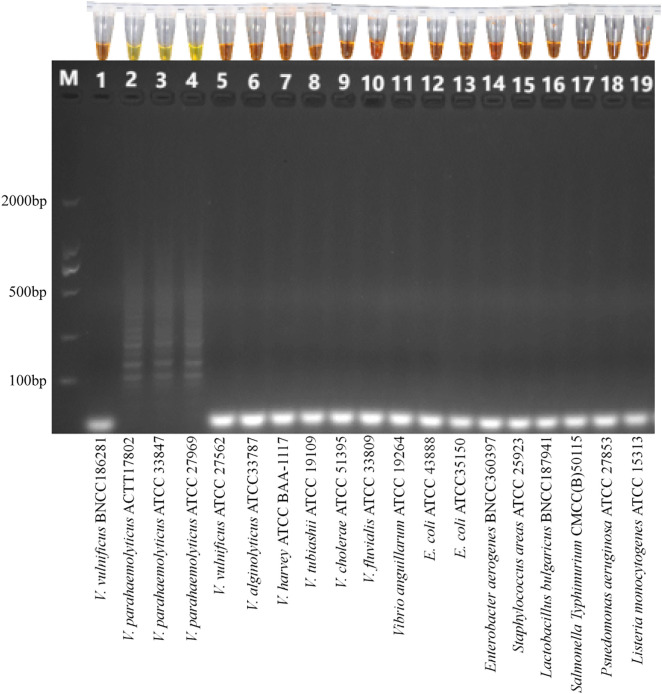
Specificity verification of the LAMP assay challenged with three *V. parahaemolyticus* strains, eight other *Vibrio* spp. strains, and eight non-*Vibrio* strains.

The salinity of samples TS1, TS3, TS4, and TS5 was adjusted to 10.00‰ using deionized water. These salinity-modified samples, along with unmodified TS2 (native salinity: 7.61‰), were inoculated with both *Vibrio* spp. and non-*Vibrio* spp. strains (final concentration approximately 10^4^ CFU/mL for each strain). Following boiling lysis, 10 µL aliquots of each sample served as DNA templates to assess the specificity of the dLAMP assay. Gel electrophoresis and visual characterizations demonstrated 100% exclusivity for non-*Vibrio* spp. strains (*n* = 8), with no false-positive or false-negative reactions observed (Fig 4). Similarly, all eight *Vibrio* spp. strains (excluding *V. parahaemolyticus*) yielded no false results. In contrast, all tested *V. parahaemolyticus* strains (ATCC 17802, ATCC 33847, and ATCC 27969) were successfully detected.

## Discussion

*Litopenaeus vannamei* aquaculture practitioners exhibit a strong desire to conveniently monitor pathogenic bacteria, such as *V. parahaemolyticus*, in culture water [3]. If boiled aquaculture water samples could directly serve as DNA templates for the LAMP assay, bypassing the need for laborious bacterial cell separation, DNA extraction, and purification steps, this approach may offer a viable pathway for developing efficient on-site detection technologies [30]. However, it has been documented that some complex substances in real and mock samples show considerable inhibitory effects on the dLAMP reaction [31–33]. Generally, *Litopenaeus vannamei* culture water exhibits complex physicochemical properties (as detailed in Table 3). Consequently, critical questions must be addressed prior to developing practical field monitoring techniques:

1. Does the addition of a defined volume (e.g., 10 µL) of such DNA template-containing aquaculture water to the LAMP reaction mixture interfere with the enzyme-catalyzed DNA amplification? If so, to what extent?
2. Does the addition compromise the interpretation of amplification results via both gel electrophoresis and visual detection methods (particularly the latter)? If so, to what extent?

Elucidating these effects is imperative for advancing field-deployable pathogen detection systems.

When extracted genomic DNA solution to provide templates, target amplicons were obtained, indicating the success of amplification. The outcome was consistent with previous report [20], thereby validating the reliability of the biochemical reaction system for the specific identification of thermolabile hemolysin *V. parahaemolyticus*.

When bacterial suspensions are boiled for an appropriate period of time, e.g., 5 min [16], DNA will be released from damaged cells [15,16]. Cell lysis simultaneously generates cell debris, denatured proteins, RNA, lipids, lipopolysaccharides, and various other organic and inorganic compounds. When these resultant mixtures served directly as DNA templates for LAMP reactions, target amplicons were obtained, characterized with both the gel electrophoresis and visualization methods. This outcome demonstrated that, as reported previously [10], the LAMP assay exhibited superior tolerance to coexisting impurities like cell debris, serum, and other organic and inorganic ingredients compared to PCR. Thus, boiled bacterial suspensions [11] and the supernatant of heated bacterial suspensions could be used directly as DNA templates for LAMP assays. Additionally, it also demonstrated that the presence of these impurities did not interfere with the characterization of the amplicons using either the gel electrophoresis or DNA dye GeneFinder™.

Purified genomic DNA obtained using the extraction kit and crude DNA obtained using the boiling method were introduced into TS1 through TS8, respectively. When 10 µL aliquots of these spiked samples were used as DNA templates for LAMP assays, amplification failed in all samples instances except for TS2 and TS3, as confirmed by gel electrophoresis or DNA dye staining. This phenomenon indicated that certain physicochemical factor(s) originating from the complex aquaculture water significantly inhibited the LAMP reaction. Previous studies on dLAMP assays were conducted primarily using bacteria suspended in simple laboratory media (e.g., water, buffer solutions, culture media) [12,13,15]. Evidence has shown that boiled bacterial suspensions and the supernatant of boiled bacterial suspensions can be used directly as DNA templates for LAMP assays [12,13]. Herein we found that not all boiled aquaculture water can be directly served as DNA templates for LAMP assays; rather, its suitability is contingent upon the intrinsic physicochemical properties of the water itself. Characterization results obtained via gel electrophoresis and visualization methods were in good consistency, suggesting that the complex physicochemical factor(s) originating from aquaculture water did not noticeably affect the detection of amplicons by visualization.

Sun et al. [16] systematically compared four DNA preparation methods—boiling, boiling in 1% Triton X-100, 0.02 M NaOH treatment, and DNAzol extraction—for LAMP assays. Their findings demonstrated superior sensitivity of the boiling method relative to alternative approaches. Nevertheless, subsequent research indicates that LAMP-based bacterial detection remains susceptible to interference by complex matrices [34]. Such inhibitors may compromise amplification efficiency through polymerase activity suppression or direct interaction with target DNA. Critically, even minor perturbations in polymerase function can significantly impact assay sensitivity and robustness [35]. Jevtuševskaja et al. [36] observed no inhibitory effects on *Bst* DNA polymerase activity from physiologically relevant concentrations of BSA, Mg² ⁺ , urea, or varied pH conditions. NaCl was found to markedly suppress amplification via enzymatic inhibition. This aligns with Tanner et al. [37], who documented broad pH tolerance (pH 6.0–10.0) across multiple *Bst* polymerase variants (*Bst* DNA polymerase, Large Fragment *Bst* DNA polymerase, and *Bst* 2.0 DNA polymerase). Notably, no studies to date have investigated the influence of key environmental parameters, including COD, DIN, TSS, and SRP, on LAMP reactions. Given that humic acid (a known COD constituent) may confound LAMP results [35], the potential interference of these uncharacterized factors warrants rigorous examination.

This study employed Pearson correlation analysis to identify elevated salinity, originating from aquaculture effluent, as the principal inhibitory factor for LAMP reactions. In contrast, no statistically significant influence was observed for other physicochemical parameters (pH, COD, TSS, SRP, and DIN). It should be noted that while the eight aquaculture water matrices encompass a broad range of physicochemical parameters, the relatively small sample size (*n* = 8) may constrain the statistical power of the correlation analysis. Future studies with a larger number of samples are warranted to further validate these findings.

To empirically validate this finding, sample TS2 was systematically adjusted to salinity gradients of 10.00‰, 13.00‰, 16.00‰, 19.00‰, 21.00‰, and 24.00‰ via NaCl supplementation, while all other parameters remained constant. 10 µL aliquots of each salinity-adjusted sample served as carriers for either extracted genomic DNA or crude DNA (prepared by boiling method) in subsequent LAMP reactions. Both electrophoretic analysis and visual inspection confirmed successful DNA amplification at salinities ≤ 19.00‰, whereas complete amplification failure occurred consistently at ≥ 21.00‰. These results demonstrate that elevated salinity significantly inhibits LAMP reactions [36]. To further corroborate this conclusion, six high-salinity samples (TS1, TS4–TS8) were diluted 1:1 with deionized water prior to use as DNA carriers. Amplicons were detected in all diluted samples, confirming salinity as a critical inhibitory factor. Additionally, analogous sample adjustment methods were applied to systematically evaluate the effects of pH (6.98–8.41), COD (6.00–12.25 mg/L), SRP (0.13–1.24 mg/L), and DIN (0.67–3.09 mg/L). Results verified that none of these parameters exerted detectable influence on LAMP amplification efficacy within the tested ranges. To ensure field applicability, centrifugation was omitted in this protocol. While particulate matter might introduce matrix heterogeneity, our results indicated that TSS levels up to 737 mg/L did not significantly interfere with the assay. In practice, thorough manual mixing after boiling helps maintain sample homogeneity, ensuring reliable detection even in complex water matrices.

*V. parahaemolyticus* cells at varying concentrations were spiked into salinity-adjusted TS2 samples (with salinities 10.00‰, 13.00‰, 16.00‰, and 19.00‰). After boiling, these mixtures served as DNA templates for LAMP assays. Visualization analysis yielded the following observations: (1) False-negative results occurred at a statistically expected level of 1^00^ CFU/mL and 10^1^ CFU/mL; (2) At 10^2^ CFU/mL, false negatives occurred when salinity ≥ 10.00‰; (3) At 10^3^ CFU/mL, false negatives occurred similarly at salinity ≥ 16.00‰; (4) At 10^4^ CFU/mL, false negatives required salinity ≥ 19.00‰; while (5) Positive detection was achieved at 10^5^ CFU/mL across all salinities, including 19.00‰. Collectively, these data demonstrate that elevated salinity reduces the sensitivity of LAMP assays for *V. parahaemolyticus*, consistent with Jevtuševskaja et al. [36]. In samples with salinity exceeding 20.00‰, the increased ionic strength likely interferes with the strand-displacement activity of the *Bst* DNA polymerase, leading to a marked decrease in sensitivity and an increased probability of false negatives at low bacterial concentrations. However, salinity ≤ 10.00‰ exerted no detectable impact on sensitivity herein. Despite generally lower sensitivity compared to conventional PCR or extracted DNA-based LAMP [38], this dLAMP assay achieved a detection limit of 10^2^ CFU/mL under optimal conditions, demonstrating a sensitivity comparable to the LAMP assay for *Listeria monocytogenes* (LOD = 6 CFU/tube; [39]). According to a previous report [40], the concentration of *V. parahaemolyticus* in shrimp aquaculture water is generally around 10^2^–10^4^ CFU/mL. Therefore, the sensitivity of this dLAMP method is sufficient to meet the practical requirements for non-quantitative screening of this pathogen in aquaculture production.

The specificity of the LAMP assay is critical for its practical application. To evaluate the specificity of this dLAMP assay, crude DNA obtained by the boiling method was loaded into carriers including TS2 (natural salinity: 7.61‰) and salinity-adjusted TS1, TS3, TS4, and TS5 (10.00‰). Visual detection of amplicons showed positive results exclusively for *V. parahaemolyticus* strains (*n* = 3), whereas all non-*Vibrio* strains (*n* = 8, encompassing Gram-positive and Gram-negative bacteria) and other *Vibrio* species (*n* = 8) yielded negative results. The absence of cross-amplification confirms the method’s high specificity under the tested experimental conditions.

**Fig 5:**
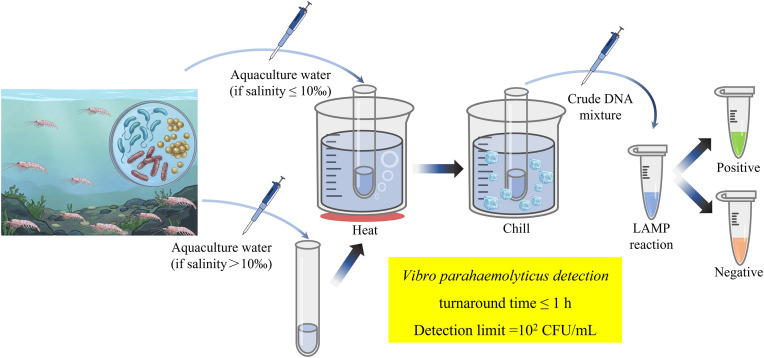
Procedure and feature of the rapid detection of *V. parahaemolyticus* using boiled aquaculture water as DNA templates for LAMP.

As illustrated in Fig 5, the dLAMP assay employs boiled aquaculture water samples as DNA templates. This streamlined protocol involves only a few steps: sampling, salinity adjustment (if necessary), boiling, chilling, and isothermal incubation. The entire procedure is completed within 1 h and requires no specialized equipment beyond a portable heating device, rendering it suitable for point-of-care applications. Although reliable for rapidly detecting *V. parahaemolyticus* in aquaculture water, this method faces notable challenges. Specifically, the molecular mechanisms by which the salinity originating from the aquaculture water influences the dLAMP reaction remain incompletely understood. Previous studies have documented that common inhibitors (e.g., calcium chloride, hematin, bile salts, humic acid, immunoglobulin G, tannic acid, and urea) systematically delay amplicon formation [35]. Consequently, further investigation is warranted to determine whether physicochemical factors in aquaculture water, particularly salinity, affect the accuracy of quantitative detection.

Despite the successful development of the dLAMP assay for *V. parahaemolyticus* detection in aquaculture water, several limitations remain to be addressed. First, while the inhibitory effect of high salinity was identified and mitigated through simple dilution, the precise molecular mechanisms by which salinity modulates *Bst* DNA polymerase efficiency and dLAMP kinetics have not yet been fully elucidated. Second, the current protocol primarily focuses on qualitative, visual endpoint detection. Although this approach is ideal for rapid on-site screening, its capacity for precise quantitative measurement is limited. Finally, while we evaluated several common physicochemical factors, the potential matrix interference from extremely high concentrations of organic matter or specific chemical additives used in diverse aquaculture species beyond *Litopenaeus vannamei* remains to be further investigated to ensure broader applicability of the dLAMP system.

## Conclusion

Extraction and purification of DNA from environmental samples are often unavailable and time-consuming in the field [30]. To address this limitation, we evaluated the suitability of boiled aquaculture water as DNA templates for LAMP assays. Water samples from eight *Litopenaeus vannamei* aquaculture systems exhibited the following parameter ranges: salinity (7.61‰–32.88‰), pH (6.98–8.41), COD (6.00–12.25 mg/L), SRP (0.13–1.24 mg/L), DIN (0.67–3.09 mg/L), and TSS (133–737 mg/L). When boiled water samples containing *V. parahaemolyticus* served as LAMP templates, only elevated salinity originating from aquaculture water exerted non-negligible effects on assay sensitivity. Following salinity adjustment to ≤ 10.00‰ with deionized water, this dLAMP assay achieved a detection limit of 10^2^ CFU/mL (with a detection probability of 67%), while maintaining high specificity. By employing DNA dye GeneFinder™ for visual endpoint detection, the entire analytical procedure, including sampling, boiling, chilling, and isothermal incubation, was accomplished within 1 h. Requiring no specialized equipment beyond a portable heating device, this protocol demonstrates significant potential for point-of-care applications, facilitating the early warning of pathogen outbreaks. It should be noted, however, that the reliability of this dLAMP-based early warning system is contingent upon salinity adjustment. In many real-world settings with high-salinity water, pre-regulating salinity to ≤ 10.00‰ remains a necessary precondition to ensure optimal assay sensitivity. Our future work will focus on elucidating the molecular mechanism through which salinity modulates LAMP efficiency and investigating the impact patterns of these physicochemical factors derived from aquaculture water (e.g., salinity) on quantitative measurements.

## Supporting information

S1 Raw Images Original, uncropped and unadjusted images underlying all staining and gel results reported in Fig 1, 3 and 4. (PDF)
