# Supplementary material for: Direct LAMP for on-site rapid detection of Vibrio parahaemolyticus in shrimp (Litopenaeus vannamei) aquaculture water
Source: PLoS One. 2026 Apr 29;21(4):e0348231. doi: 10.1371/journal.pone.0348231 (PMC13127935; doi:10.1371/journal.pone.0348231)

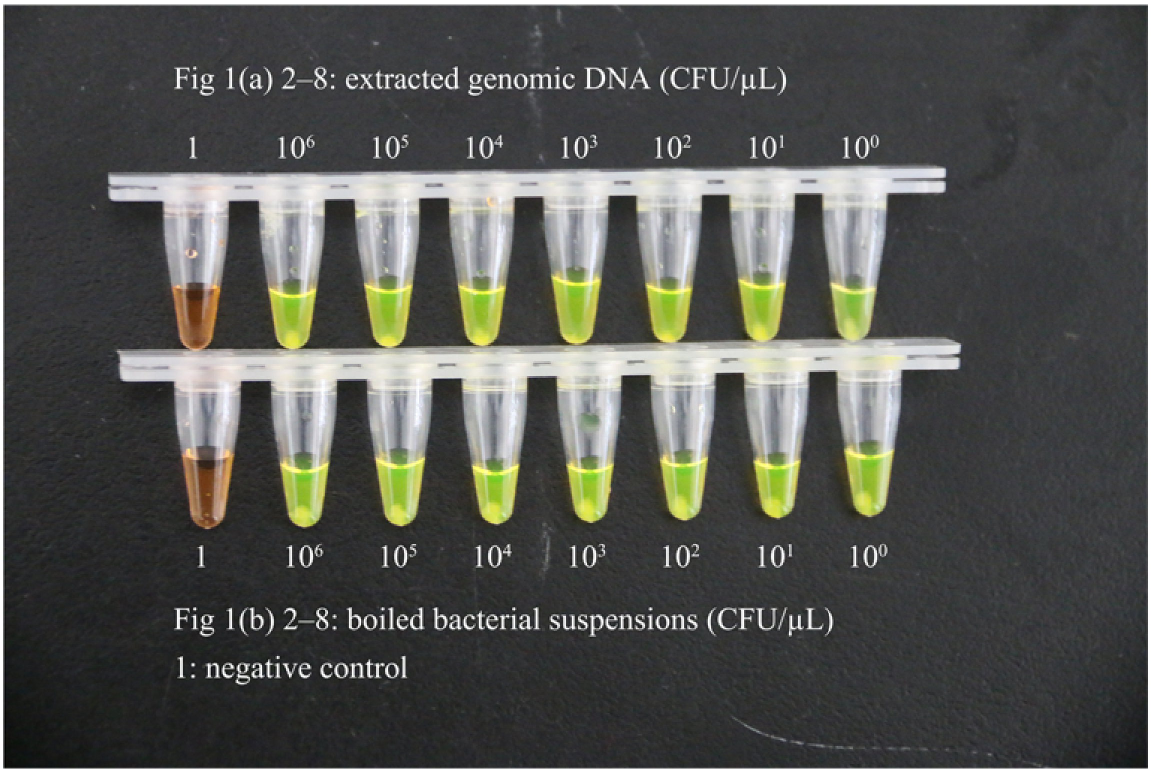
Fig 1(a) 2–8: extracted genomic DNA (CFU/μL)
1 10⁶ 10⁵ 10⁴ 10³ 10² 10¹ 10⁰
1 10⁶ 10⁵ 10⁴ 10³ 10² 10¹ 10⁰
Fig 1(b) 2–8: boiled bacterial suspensions (CFU/μL)
1: negative control

Fig 1(b)

Fig 1(a)

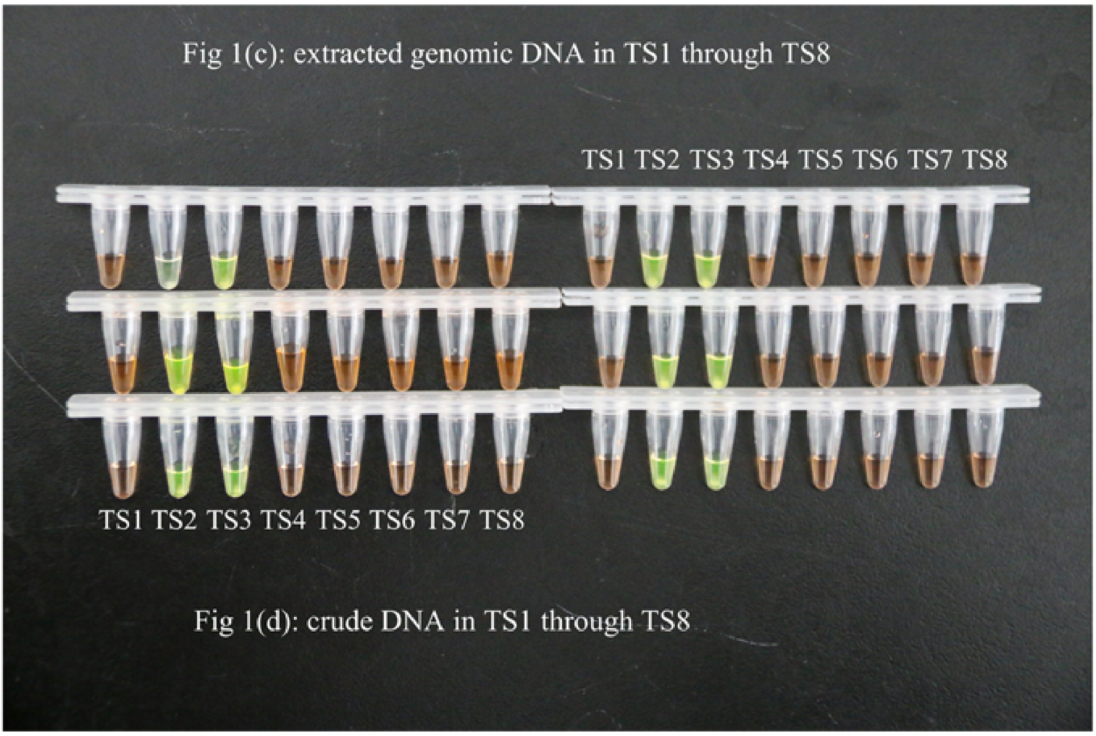

X
X
Fig 1(c)

Fig 1(d)
X
X

Fig 1(a)

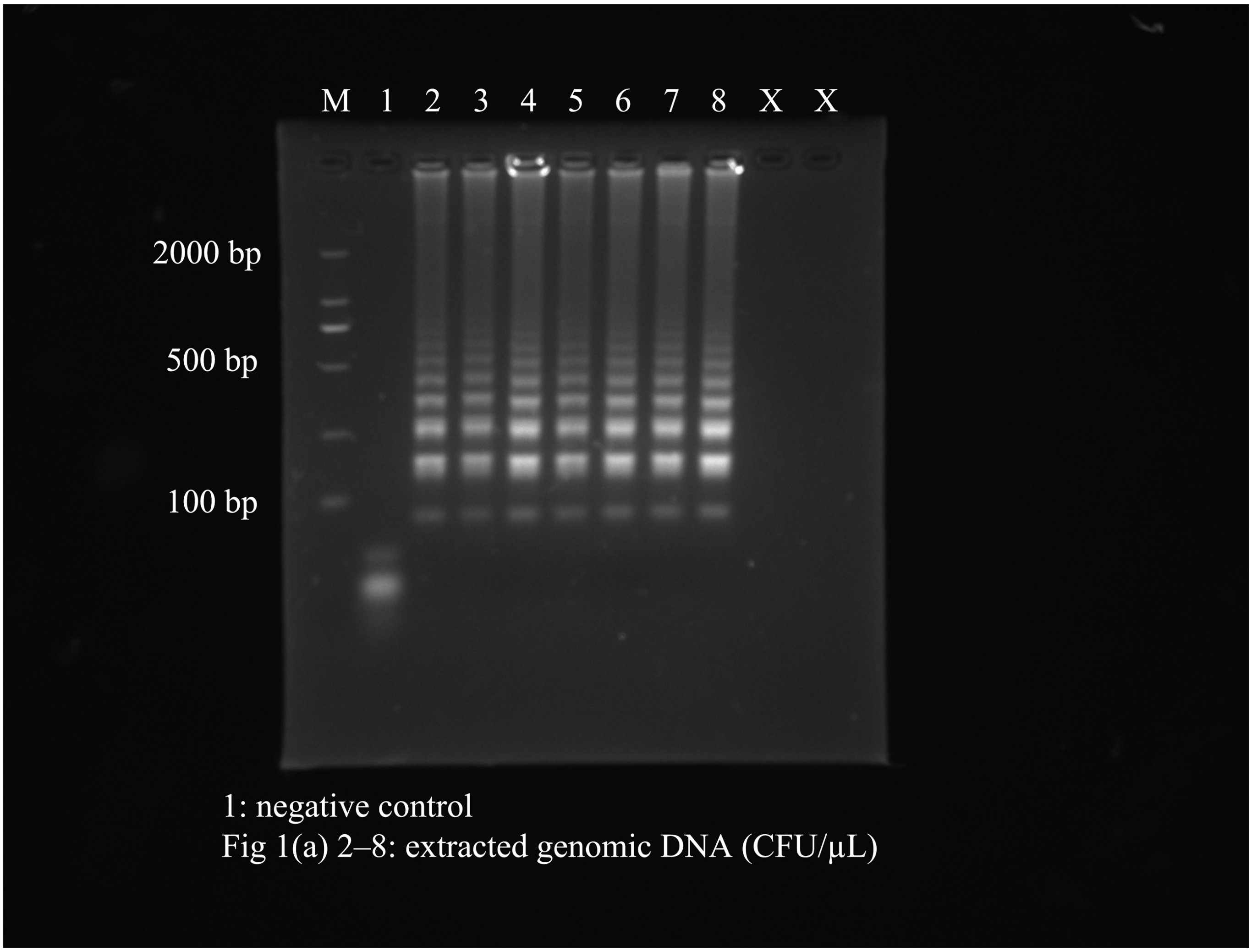

1: negative control
Fig 1(a) 2–8: extracted genomic DNA (CFU/μL)

Fig 1(b)

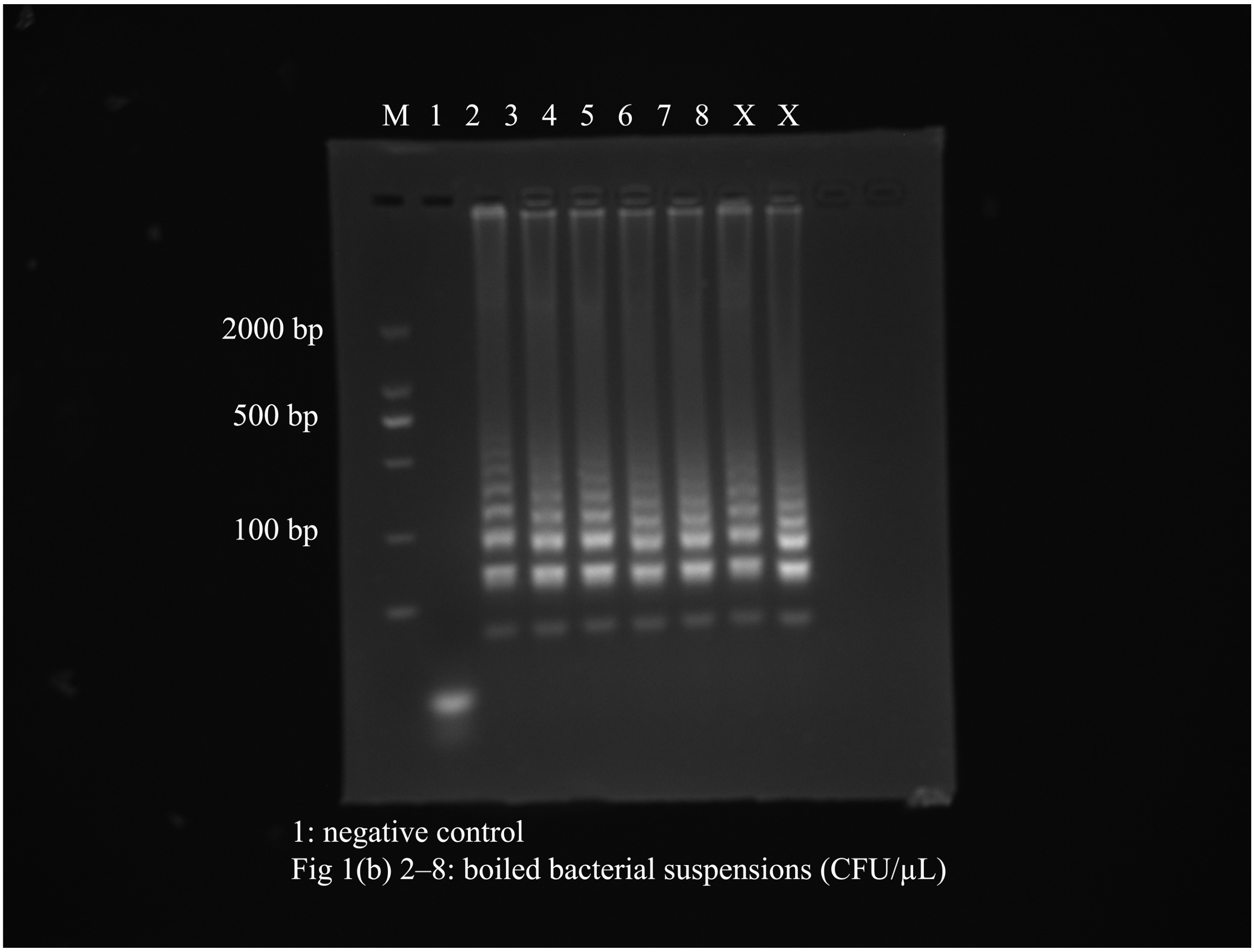

1: negative control
Fig 1(b) 2–8: boiled bacterial suspensions (CFU/µL)

Fig 1(c)

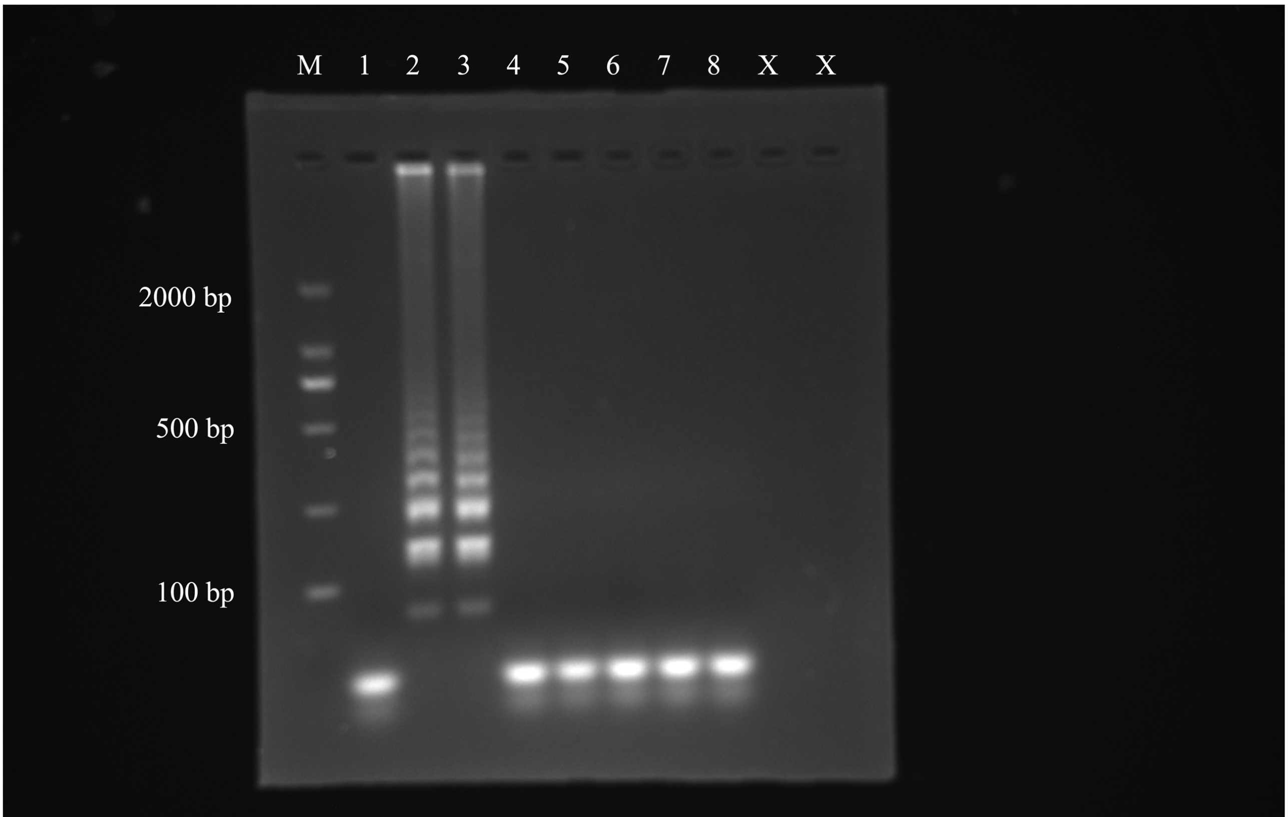

Fig 1(c).1-8: extracted genomic DNA in TS1 through TS8

Fig 1(d)

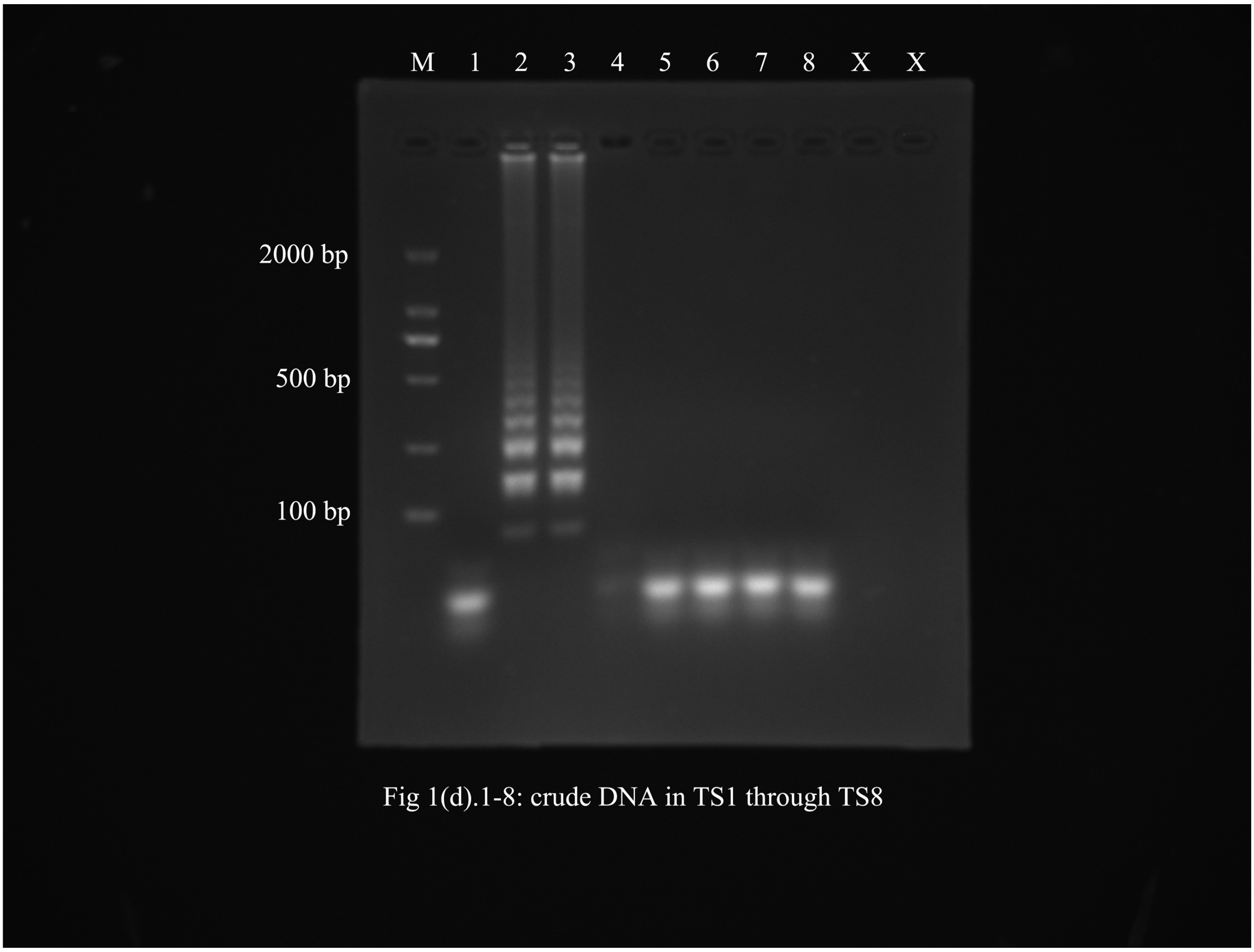

Fig 1(d).1-8: crude DNA in TS1 through TS8

Fig 3(a)

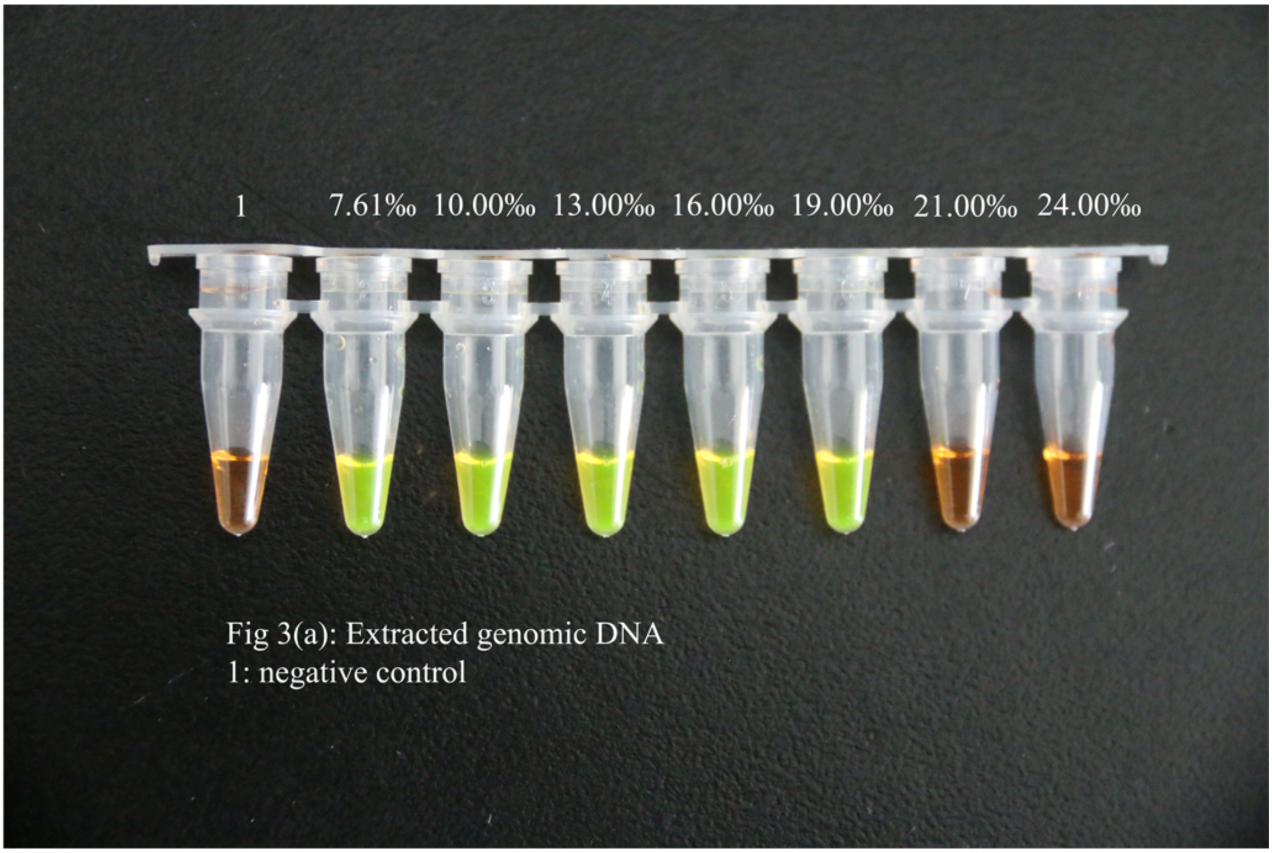
1
7.61‰
10.00‰
13.00‰
16.00‰
19.00‰
21.00‰
24.00‰
Fig 3(a): Extracted genomic DNA
1: negative control

Fig 3(b)

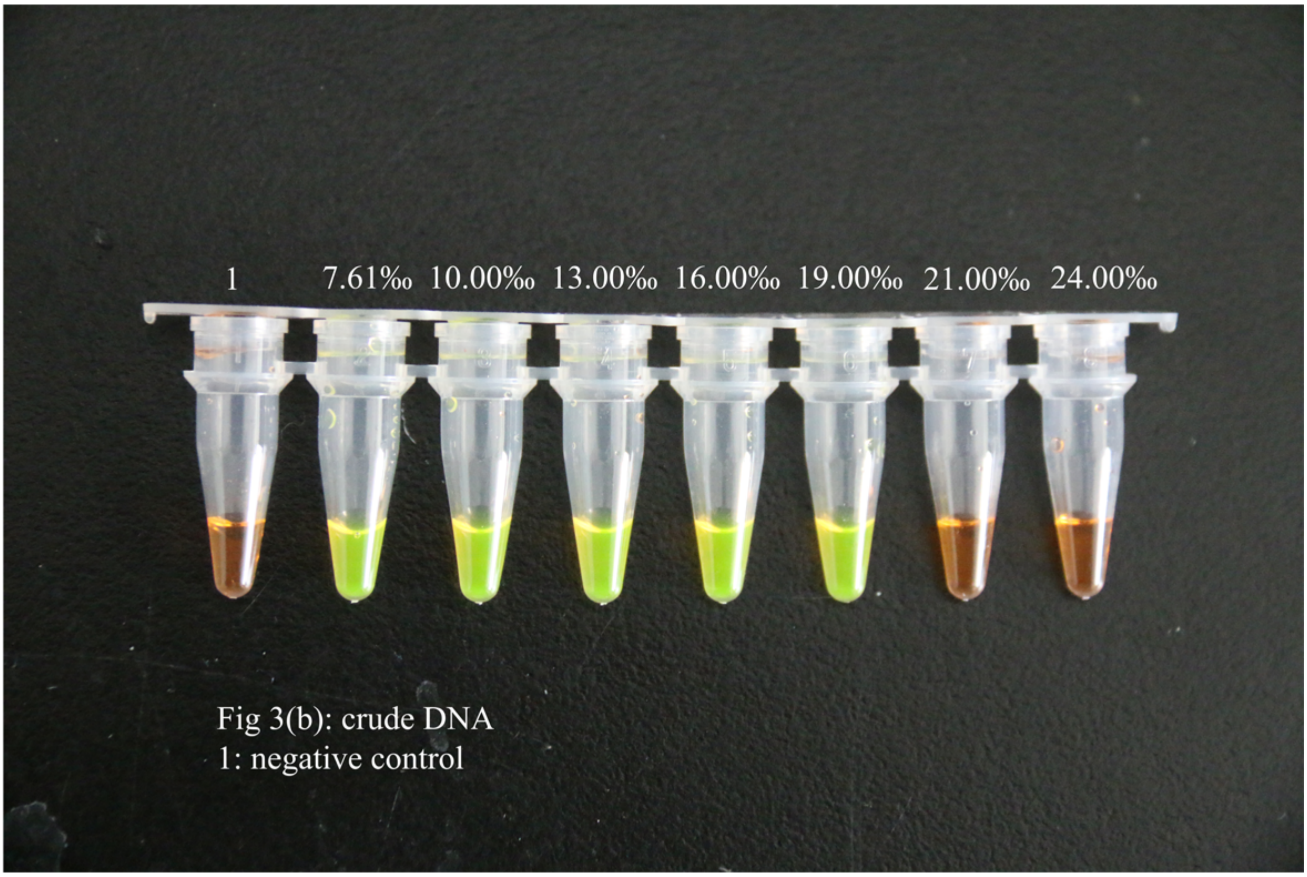
1 7.61‰ 10.00‰ 13.00‰ 16.00‰ 19.00‰ 21.00‰ 24.00‰
Fig 3(b): crude DNA
1: negative control

Fig 3(a)

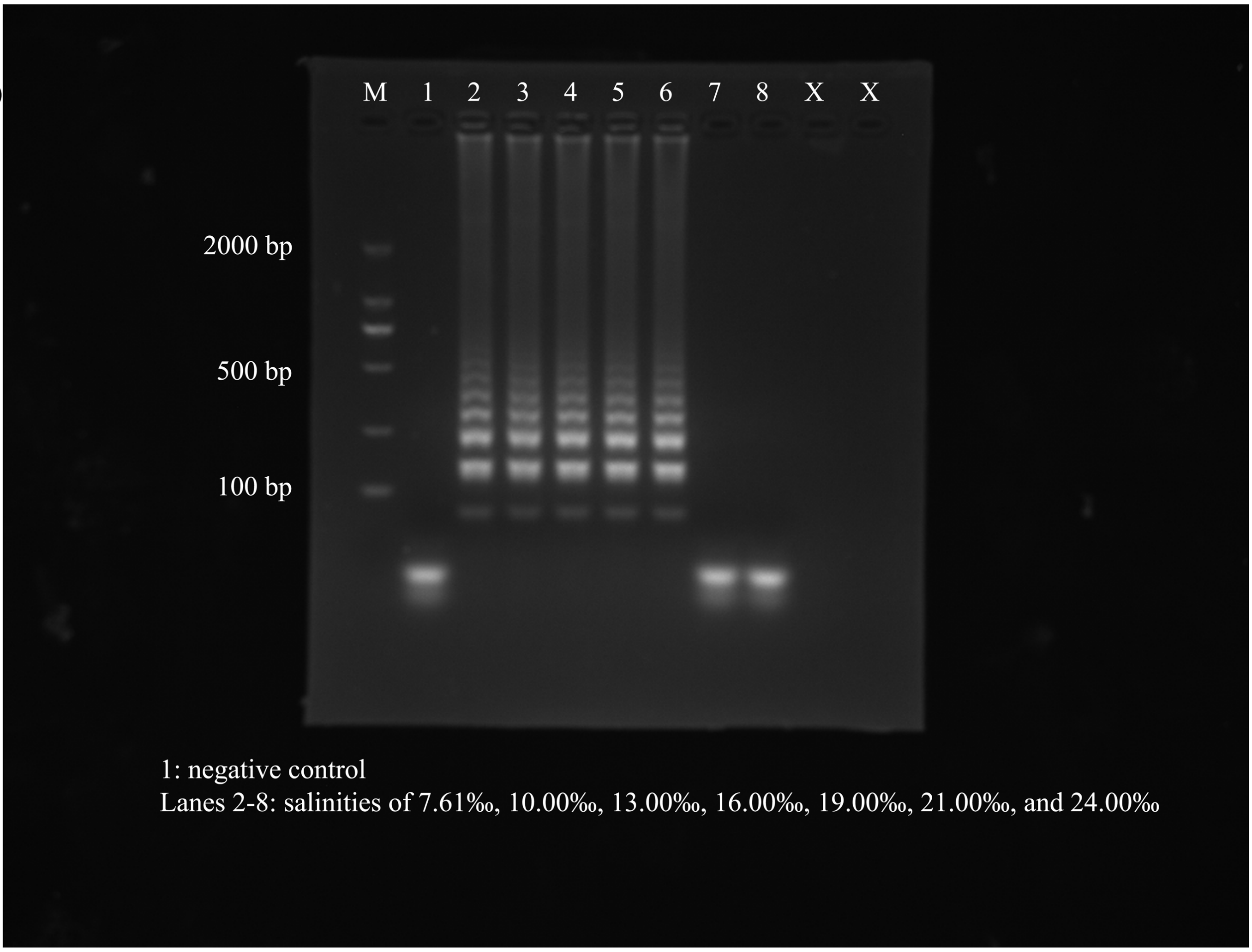
M 1 2 3 4 5 6 7 8 X X
2000 bp
500 bp
100 bp
1: negative control
Lanes 2-8: salinities of 7.61‰, 10.00‰, 13.00‰, 16.00‰, 19.00‰, 21.00‰, and 24.00‰

Fig 3(b)

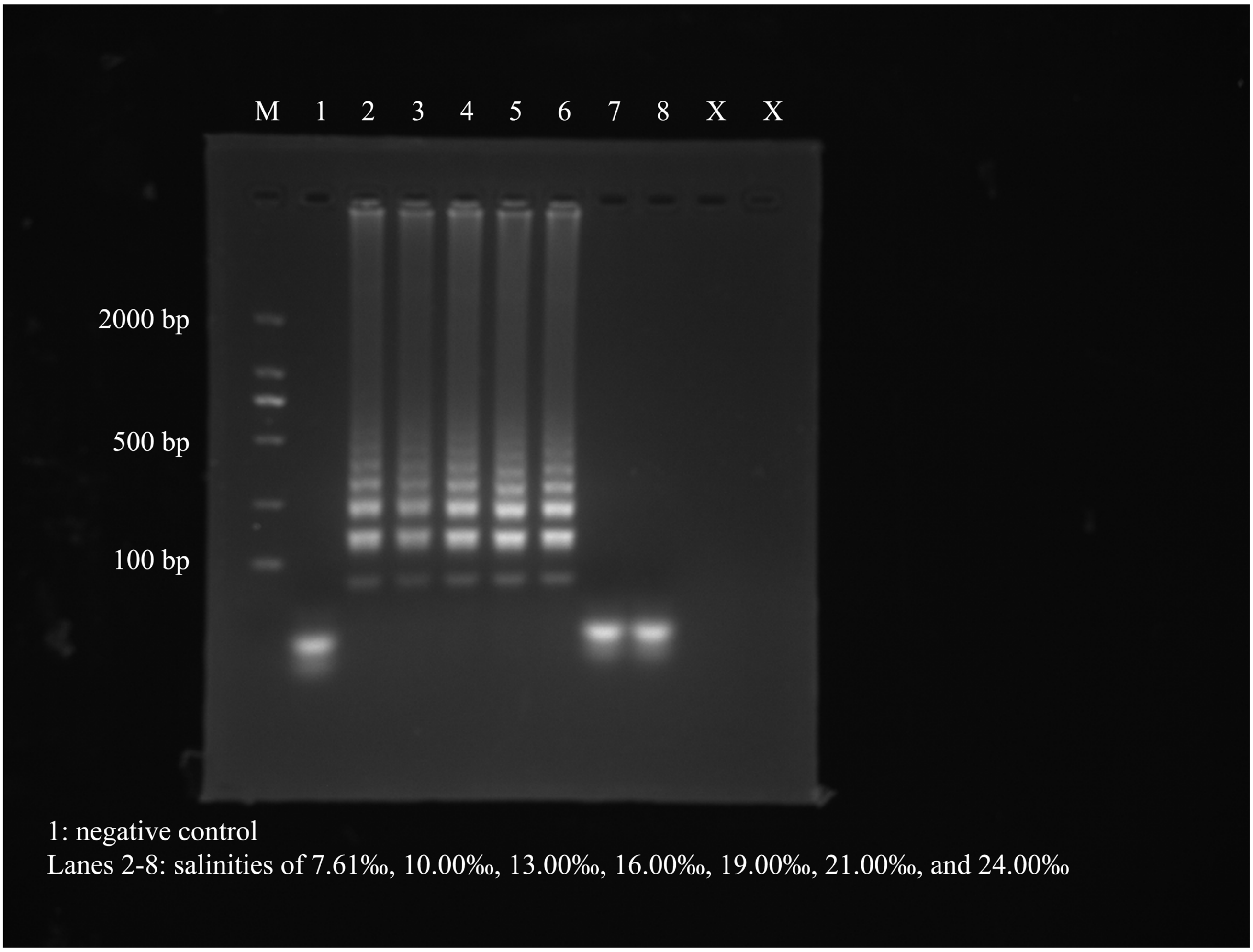
M 1 2 3 4 5 6 7 8 X X
2000 bp
500 bp
100 bp
1: negative control
Lanes 2-8: salinities of 7.61‰, 10.00‰, 13.00‰, 16.00‰, 19.00‰, 21.00‰, and 24.00‰

Fig 4

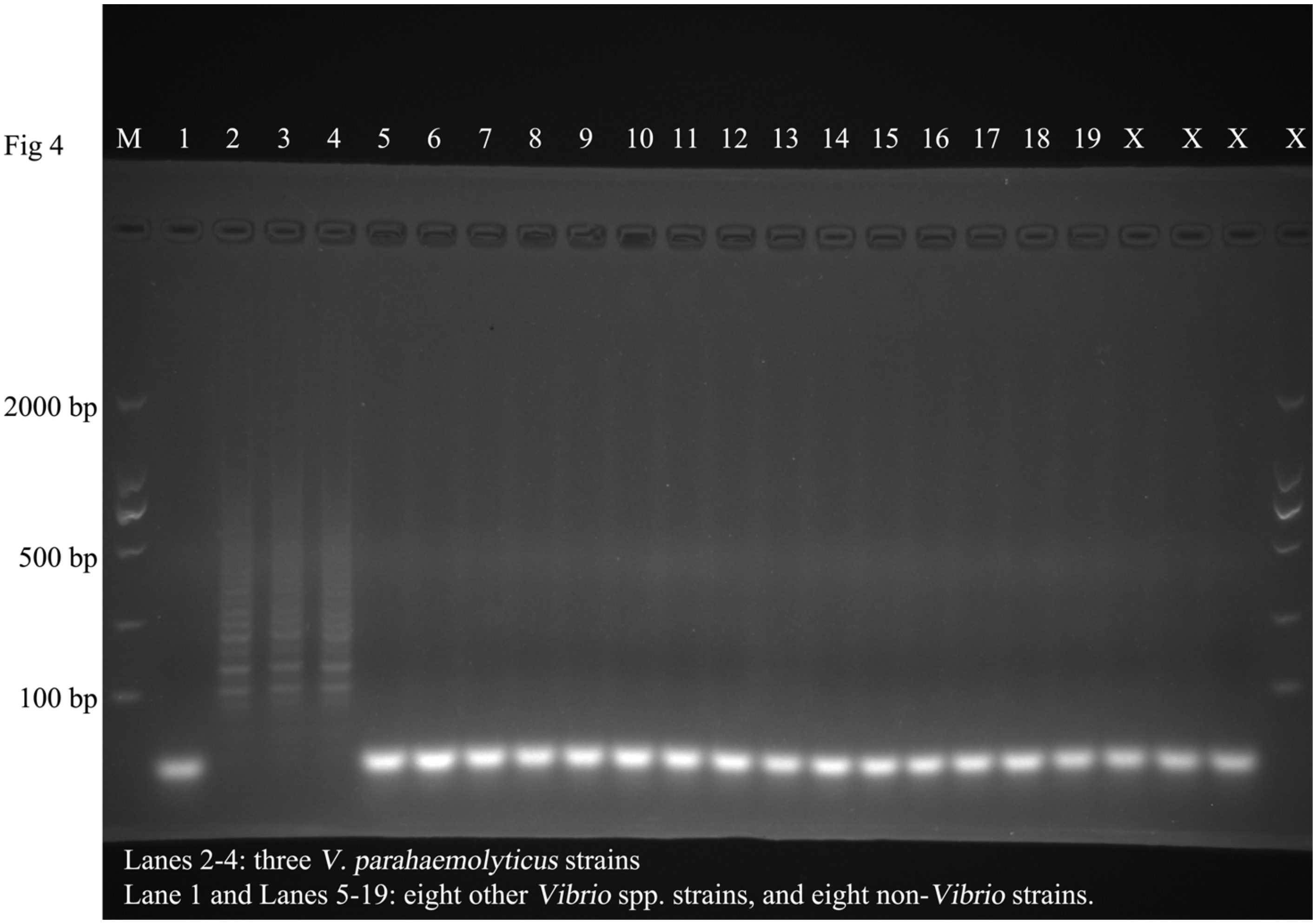

Lanes 2-4: three *V. parahaemolyticus* strains
Lane 1 and Lanes 5-19: eight other *Vibrio* spp. strains, and eight non-*Vibrio* strains.

Fig 4

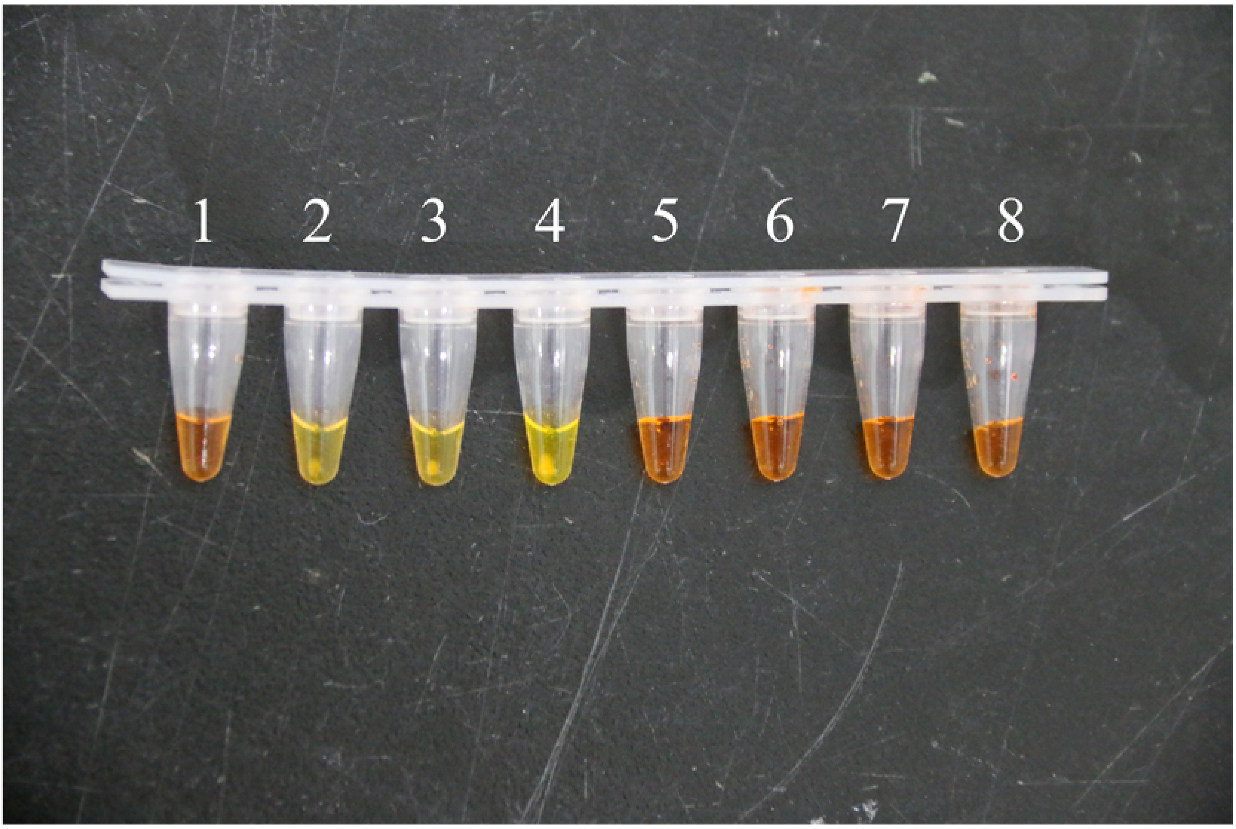

Fig 4

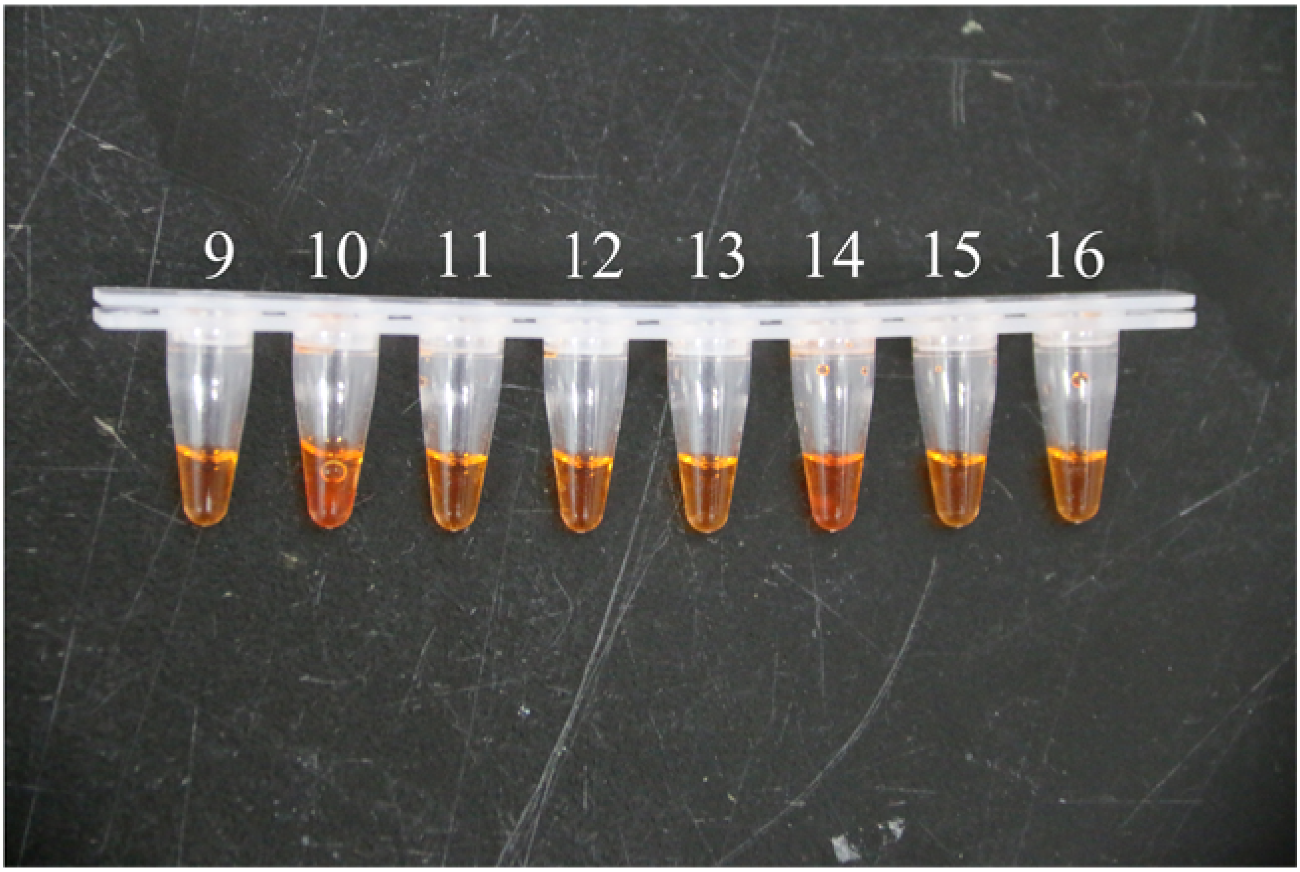

Fig 4

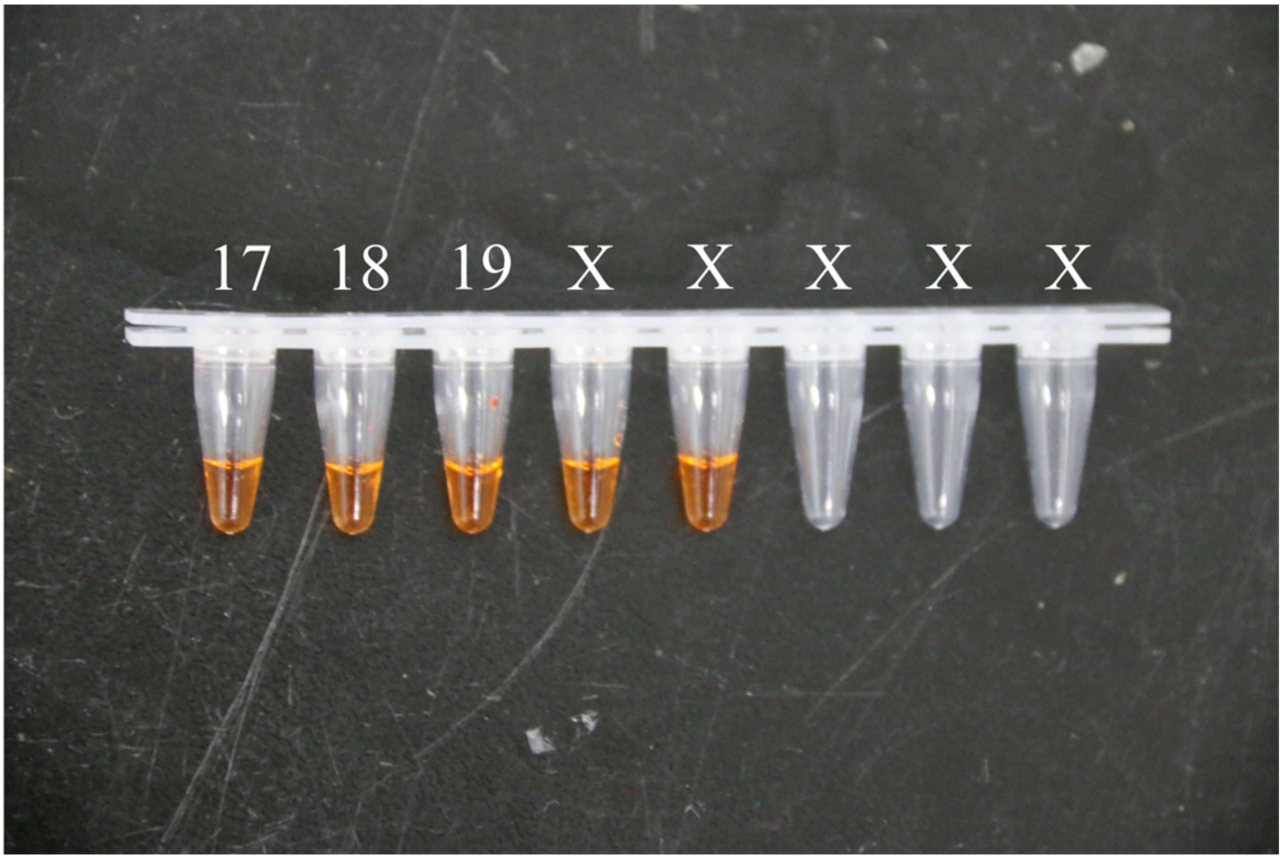

Supplement: S1 Raw Images — (PDF) [file pone.0348231.s001.pdf]
